# Emerging Burden of Scrub Typhus: A Comprehensive Analysis of Clinical, Demographic, and Occupational Risk Factors in a Tertiary Care Center in Rajasthan, India

**DOI:** 10.7759/cureus.100796

**Published:** 2026-01-05

**Authors:** Pinky Sherawat, Jitendra Panda, Shiv Prakash Sharma, Nilofer Khayyam

**Affiliations:** 1 Microbiology, Rajasthan University of Health Sciences (RUHS) College of Medical Sciences (CMS), Jaipur, IND; 2 Preventive Medicine, Rajasthan University of Health Sciences (RUHS) College of Medical Sciences (CMS), Jaipur, IND

**Keywords:** clinical profile, epidemiology, occupational risk, rajasthan, scrub typhus, zoonosis

## Abstract

Introduction

Scrub typhus, caused by *Orientia tsutsugamushi*, is a re-emerging public health threat in India. The detailed characterization of its local epidemiology and clinical presentation is essential for timely diagnosis and effective public health intervention.

Methods

A cross-sectional, observational study was conducted from January to April 2024. A total of 156 patients with acute undifferentiated febrile illness were screened, of which 46 IgM enzyme-linked immunosorbent assay (ELISA)-confirmed scrub typhus cases were included for analysis. Data on demographic characteristics, geographical distribution, occupation, clinical features, and laboratory parameters were systematically collected and analyzed.

Results

Scrub typhus was the leading cause of acute undifferentiated febrile illnesses (AUFI) (46/156, 29.5%), showing a significant predilection for women (29, 63.0%), rural residents (32, 69.6%), and agricultural workers (25, 54.3%), with a distinct seasonal peak in March-April (39, 84.8%). The most common symptoms were fever (46, 100%), myalgia (30, 65.2%), and headache (28, 60.9%); notably, an eschar was absent in all patients. Thrombocytopenia was significantly more prevalent in hospitalized (inpatient department {IPD}) patients compared to outpatients (outpatient department {OPD}) (five, 45.5%, versus four, 11.4%; p=0.010).

Conclusion

The distinct demographic profile and frequent hepatic dysfunction, in the absence of an eschar, are critical features that should guide early diagnosis and empiric treatment in this endemic region. Public health efforts should target at-risk populations through awareness and vector control measures.

## Introduction

Scrub typhus, a potentially fatal zoonotic disease caused by the bacterium *Orientia tsutsugamushi*, poses a significant and re-emerging health challenge across the Tsutsugamushi Triangle, which encompasses large parts of Asia, including India [1]. The disease is transmitted to humans through the bite of infected chigger mites (*Leptotrombidium *species), with rodents serving as the primary reservoir hosts [2]. In recent years, India has witnessed a dramatic surge in scrub typhus cases, accounting for a substantial proportion of acute undifferentiated febrile illnesses (AUFI) [3].

The clinical presentation of scrub typhus is notoriously nonspecific, often mimicking other common febrile illnesses such as dengue, leptospirosis, and typhoid, which frequently leads to misdiagnosis and delays in initiating appropriate antibiotic therapy [4]. While the presence of an eschar, a localized necrotic lesion at the site of the chigger bite, is considered a pathognomonic sign, its reported prevalence is highly variable, and it is absent in a significant number of cases, further complicating clinical diagnosis [5]. The variability in clinical manifestations and the lack of a reliable clinical signpost underscore the importance of understanding the region-specific epidemiological and clinical profile of the disease.

Rajasthan, a state in northwestern India, has reported numerous outbreaks and sporadic cases of scrub typhus, yet comprehensive data from this region remain limited [6]. A detailed understanding of the local demographic patterns, seasonal trends, occupational risks, and clinical features is crucial for developing a high index of suspicion among healthcare providers, facilitating early diagnosis, and formulating targeted public health interventions. This study aimed to comprehensively characterize the clinical and epidemiological profile of patients with scrub typhus presenting to a tertiary care hospital in Jaipur, Rajasthan.

## Materials and methods

Study design and population

A laboratory-based, cross-sectional study was conducted over four months from January to April 2024 at the Department of Microbiology, Rajasthan University of Health Sciences (RUHS) College of Medical Sciences, Jaipur, India. The study was approved by the RUHS College of Medical Sciences Ethics Committee (RUHS-CMS/Ethics Comm./2023/288).

Inclusion Criteria

Patients of all age groups and both sexes presenting with an acute undifferentiated fever (axillary temperature of ≥100.4°F/38°C) of less than 14 days were included.

Acute undifferentiated febrile illness (AUFI) was defined as a documented acute fever (axillary temperature of ≥100.4°F/38°C) of less than 14 days, without any obvious focus of infection at the time of presentation.

Exclusion Criteria

Patients who did not provide written informed consent were excluded. A total of 156 eligible patients were enrolled after obtaining written informed consent.

Study objectives

The objectives of this study were to estimate the burden of scrub typhus among patients presenting with acute undifferentiated febrile illness (AUFI) at a tertiary care center; to characterize the demographic, geographical, and occupational profile of patients with confirmed scrub typhus; and to describe the clinical and laboratory features of scrub typhus in this cohort and compare clinical and laboratory parameters between outpatient (outpatient department {OPD}) and inpatient (inpatient department {IPD}) groups to identify markers associated with disease severity.

Laboratory confirmation and data collection

Scrub typhus infection was confirmed using the Scrub Typhus Detect™ IgM enzyme-linked immunosorbent assay (ELISA) system (InBios International, Inc., Seattle, WA) according to the manufacturer's protocol, with an optical density (OD) of ≥0.5 considered positive. For the 46 patients with confirmed scrub typhus, detailed data were extracted from a structured proforma, including demographic details (age, gender, and residence), occupation, month of diagnosis, clinical symptoms at presentation, and routine laboratory investigations such as complete blood count, liver function tests, and renal function tests.

Statistical analysis

Data were analyzed using descriptive statistics. Categorical variables were expressed as frequencies (n) and percentages (%). The chi-square test was used to compare clinical and laboratory parameters between outpatient (OPD) and inpatient (IPD) groups. A p-value of less than 0.05 was considered statistically significant. Analysis was performed using SPSS Statistics version 28.0 (IBM Corp., Armonk, NY).

## Results

During the four-month study period, 46 of the 156 febrile patients (46/156, 29.5%) were confirmed as scrub typhus cases by IgM ELISA. The cohort showed a female preponderance (29, 63.0%), with most patients belonging to the 20-39-year age group (30, 65.2%), residing in rural areas (32, 69.6%), and engaging in agricultural work (25, 54.3%). Cases peaked in March (19, 41.3%) and April (20, 43.5%). The complete demographic and epidemiological characteristics of these patients are detailed in Table 1.

**Table 1:** Demographic and Epidemiological Profile of Patients With Scrub Typhus (n=46) This table presents the baseline characteristics of the study population. Data are presented as frequencies (n) and percentages (%). No comparative statistical tests were applied, as the data describe a single cohort

Characteristic	Category	Frequency, n (%)
Gender	Male	17 (37.0)
	Female	29 (63.0)
Age group	<20 years	8 (17.4)
	20-39 years	30 (65.2)
	40-59 years	6 (13.0)
	≥60 years	2 (4.3)
Residence	Rural	32 (69.6)
	Urban	14 (30.4)
Occupation	Agriculture	25 (54.3)
	Housewife	6 (13.0)
	Labor	7 (15.2)
	Others	8 (17.4)
Month of diagnosis	January	1 (2.2)
	February	6 (13.0)
	March	19 (41.3)
	April	20 (43.5)

The clinical presentation of the 46 patients with scrub typhus is summarized in Table 2. Fever was a universal finding. Myalgia (30, 65.2%) and headache (28, 60.9%) were the most common accompanying symptoms. Notably, an eschar was not observed in any patient in this cohort.

**Table 2:** Clinical Presentation of Patients With Scrub Typhus (n=46) This table details the frequency of clinical symptoms at presentation among the 46 patients with confirmed scrub typhus. Data are presented as frequencies (n) and percentages (%). No comparative statistical tests were applied, as the data describe a single cohort

Symptom	Frequency, n (%)
Fever	46 (100.0)
Myalgia	30 (65.2)
Headache	28 (60.9)
Arthralgia	25 (54.3)
Cough	21 (45.7)
Abdominal pain	15 (32.6)
Nausea and vomiting	12 (26.1)
Lymphadenopathy	8 (17.4)
Rash	6 (13.0)
Hepatosplenomegaly	3 (6.5)
Eschar	0 (0.0)

Key laboratory findings are presented in Table 3. Hematological abnormalities included anemia (15, 32.6%) and thrombocytopenia (nine, 19.6%). The most striking biochemical abnormality was elevated direct bilirubin, observed in 19 (41.3%) patients.

**Table 3:** Laboratory Findings in Patients With Scrub Typhus (n=46) This table summarizes the hematological and biochemical abnormalities observed in the 46 patients with confirmed scrub typhus. Data are presented as frequencies (n) and percentages (%). No comparative statistical tests were applied, as the data describe a single cohort

Parameter	Abnormality	Frequency, n (%)
Hematology	Anemia (Hb<11 g/dL)	15 (32.6)
	Leukocytosis (>11,500 cells/mm³)	12 (26.1)
	Thrombocytopenia (<150,000 cells/mm³)	9 (19.6)
Liver function tests	Elevated direct bilirubin (>3 mg/dL)	19 (41.3)
	Elevated total bilirubin (>1.1 mg/dL)	6 (13.0)
	Elevated hepatic transaminases	7 (15.2)
	Hypoalbuminemia (<2.5 g/dL)	3 (6.5)
Renal function tests	Elevated serum urea (>50 mg/dL)	3 (6.5)
	Elevated serum creatinine (>1.5 mg/dL)	1 (2.2)

A comparison of clinical and laboratory parameters between the 35 outpatients (OPD) and 11 inpatients (IPD) revealed significant findings, as detailed in Table 4. Thrombocytopenia was significantly more prevalent among hospitalized patients (five, 45.5%) compared to outpatients (four, 11.4%; p=0.010).

**Table 4:** Comparison of Clinical and Laboratory Features Between OPD and IPD Patients With Scrub Typhus This table compares features between outpatient (OPD) and inpatient (IPD) patients with scrub typhus using the chi-square test. A p-value of <0.05 was considered statistically significant. The significant p-value is denoted with "*". Thrombocytopenia was significantly more frequent in IPD patients OPD, outpatient department; IPD, inpatient department

Characteristic		OPD (n=35)	IPD (n=11)	Test statistic (χ²)	P-value
Clinical features	Headache	22 (62.9%)	6 (54.5%)	0.24	0.623
	Myalgia	24 (68.6%)	6 (54.5%)	0.75	0.387
	Abdominal pain	9 (25.7%)	6 (54.5%)	3.21	0.073
	Nausea/vomiting	7 (20.0%)	5 (45.5%)	2.76	0.097
	Rash	3 (8.6%)	3 (27.3%)	2.60	0.107
Laboratory features	Thrombocytopenia	4 (11.4%)	5 (45.5%)	6.58	0.010*
	Elevated direct bilirubin	12 (34.3%)	7 (63.6%)	2.95	0.086
	Hypoalbuminemia	1 (2.9%)	2 (18.2%)	3.27	0.070

## Discussion

This study provides a detailed snapshot of the clinical and epidemiological dimensions of scrub typhus in a tertiary care setting in Rajasthan, revealing several critical insights. The finding that 29 (63.0%) of the 46 patients were women aligns with several studies from India and Nepal that report a similar female preponderance [7,8]. This may be attributed to the higher risk of exposure for women engaged in agricultural activities and household work in peri-domestic environments where chigger mites may thrive.

The disease showed a strong predilection for the rural population (32, 69.6%) and individuals involved in agriculture (25, 54.3%), which is consistent with the known ecology of the vector [9]. The predominance of cases in the 20-39-year age group (30, 65.2%) likely reflects the demographic most actively involved in outdoor and agricultural work. The sharp increase in cases during March (19, 41.3%) and April (20, 43.5%) suggests a distinct seasonal peak in the warmer, drier months in this region, a pattern that can inform the timing of public health advisories and vector control measures.

A pivotal clinical finding was the complete absence of an eschar in our patient cohort (zero, 0.0%). This contrasts with reports from some other regions but is consistent with several studies from India, highlighting that the reliance on this sign for clinical diagnosis is fraught with risk and can lead to underdiagnosis [10,11]. The clinical profile was otherwise typical, with fever, myalgia, and headache being the most common symptoms.

The laboratory profile offered important clues for diagnosis. The high frequency of elevated direct bilirubin (19, 41.3%) is a notable feature, indicating significant hepatic involvement in scrub typhus, even in the absence of overt jaundice. Our comparative analysis between OPD and IPD patients provided a key marker for disease severity. Thrombocytopenia was significantly more prevalent among hospitalized patients (five, 45.5%) compared to outpatients (four, 11.4%; p=0.010). This finding is consistent with other studies identifying thrombocytopenia as a predictor of severe disease and aligns with its known pathophysiology of endothelial injury and disseminated vasculitis in scrub typhus [12,13]. Furthermore, trends suggesting a higher frequency of abdominal pain, hypoalbuminemia, and elevated direct bilirubin in IPD patients, though not statistically significant in this cohort, warrant further investigation in larger studies. It is important to note that in this study, hospitalization (IPD status) was used as a pragmatic surrogate marker for disease severity. The decision for admission was based on the treating physician's clinical judgment, which may have included factors such as high-grade persistent fever, systemic symptoms, laboratory abnormalities, or comorbidities, rather than a predefined standardized severity score. The absence of strict, protocol-driven admission criteria limits the strength of severity-related interpretations, such as the association of thrombocytopenia with more severe disease. Future prospective studies employing validated scrub typhus severity scores would provide a more robust assessment of risk factors for severe outcomes.

Limitations

This study was conducted at a single center over a relatively short duration, which may limit the generalizability of the findings across all seasons and regions. The diagnosis was based on a single serological test (IgM ELISA), and the gold standard immunofluorescence assay (IFA) or polymerase chain reaction (PCR) confirmation on all samples was not performed. The sample size, particularly for the IPD group (n=11), limits the statistical power for some comparative analyses. Furthermore, as an observational study, it identifies associations but cannot establish causality.

## Conclusions

Scrub typhus in Rajasthan predominantly affects young, rural adults, with a significant burden on women and agricultural workers. The consistent absence of an eschar necessitates that clinicians maintain a high index of suspicion based on epidemiological and laboratory clues, such as hepatic dysfunction. The identification of thrombocytopenia as a significant marker for hospitalization provides a valuable tool for risk stratification. These findings underscore the urgent need for integrated public health strategies focusing on awareness in agrarian communities and strengthening laboratory capacity for accurate diagnosis in endemic regions.
